# Paraventricular nucleus neurons: important regulators of respiratory movement in mice with chronic intermittent hypoxia

**DOI:** 10.1080/07853890.2025.2588664

**Published:** 2025-11-18

**Authors:** Linglin Liu, Junhong Zhang, Shuangshuang Song, Wu Li, Wei Xiong

**Affiliations:** Department of Geriatrics, The First Affiliated Hospital, Army Medical University, Chongqing, China

**Keywords:** CIH, PVN, OSAHS, chemogenetics, neural circuit, PVN-preBötC

## Abstract

**Objective:**

Obstructive sleep apnea-hypopnea syndrome (OSAHS) is a prevalent respiratory disorder. Chronic intermittent hypoxia (CIH) is a key pathophysiological change linked to the development of OSAHS. The paraventricular hypothalamic nucleus (PVN) plays a role in respiratory control. This study aimed to explore a novel treatment for OSAHS through PVN stimulation.

**Method:**

The Cre/LoxP gene expression strategy, along with the viral tracer technique and immunofluorescence staining, was employed to confirm the anatomical relationship between the PVN and the preBötzinger complex (preBötC) in mice. A CIH mouse model was established, and chemogenetics was integrated with whole-body plethysmography (WBP) to monitor the respiratory function changes in CIH mice.

**Result:**

(1) A notably higher density of c-Fos-positive neurons was detected in the PVN of CIH mice compared to normoxic mice (*p* < 0.0001). (2) Neuronal somata and nerve fibers that were co-labeled were found in both the PVN and preBötC. (3) Modulating PVN neurons through chemogenetic methods affected respiratory frequency (RF) and minute ventilation (MV) in CIH mice, with tidal volume (TV) remaining unchanged. Furthermore, the activation of PVN neurons increased c-Fos expression in the preBötC.

**Conclusion:**

A direct relationship exists between the PVN and preBötC. CIH increased c-Fos expression in respiratory-related nuclei, including the PVN. Modulating the PVN activity impacts MV by adjusting the RF; without influencing arterial blood gas levels. The PVN is probably involved in respiratory control *via* the PVN-preBötC circuit.

## Introduction

Obstructive sleep apnea–hypopnea syndrome(OSAHS) is a common respiratory disorder and an independent risk factor for cardiovascular, cerebrovascular, nervous system, and metabolic dysfunctions [1,2]. Chronic intermittent hypoxia (CIH) is a primary pathophysiological change associated with OSAHS [3]. The prevalence of OSAHS in patients aged 60 and above has shown a linear increase with age [4]. However, current treatments mainly involve positive-pressure ventilation, leading to low patient compliance and suboptimal therapeutic outcomes. Consequently, identifying novel therapeutic approaches beyond existing methods is a pressing issue that requires attention.

Human respiratory movement is controlled by a complex neural network dedicated to respiratory regulation [5,6]. The pre-Bötzinger complex (preBötC), situated in the ventrolateral medulla, serves as the source of inspiratory rhythms [7]. It integrates both excitatory and inhibitory inputs to produce a three-phase respiratory pattern (inspiration, post-inspiration, and expiration) and is influenced by neuromodulators [8–10]. The hypothalamus plays a vital role in regulating various autonomic brain functions, including the respiratory network [11–16]. The paraventricular nucleus (PVN) is recognized as an important regulatory nucleus in the hypothalamus due to its high-level multimodal integration functions [17]. Neurons in the PVN project to established respiratory control regions in the brainstem and spinal cord, such as the rostral ventrolateral medulla (RVLM), parabrachial complex (PBC), nucleus tractus solitarius (NTS), and the spinal phrenic nucleus [18–25]. Additionally, the PVN receives afferent fibers from various brain regions, including the NTS, the dorsal motor nucleus of the vagus (DMV), the hippocampus, and the arcuate nucleus [26,27]. This positioning highlights the PVN as a crucial connector in the respiratory neural network. Based on the potential role of the PVN in respiratory control, this study aims to investigate the existence of a direct PVN to preBötC projection and its functional significance. Specifically, we hypothesize that this projection is enhanced by CIH (manifested as increased c-Fos expression in PVN) and that activation of this PVN-preBötC circuit participates in the respiratory responses to CIH.

This study aimed to confirm the critical role of the PVN in the respiratory neural network and its regulatory function in respiration under CIH conditions using viral tracing technology, immunofluorescence staining, and chemogenetics in conjunction with whole-body plethysmography (WBP) in conscious mice. These findings may offer new insights into hypoxia-related diseases such as OSAHS.

## Materials and methods

The study commenced preparation in May 2022, with animal experiments starting in August, and continued through December 2024.

### Laboratory animals

A total of 99 100 male C57BL/6J mice (7 weeks old, weighing 18–20 g) were procured from Hunan Slack Jingda Company and were used in this study. 3 to 5 mice were housed per cage in a controlled environment maintained at a constant temperature of 25 °C with a 12-hour dark/light cycle. The mice had free access to food and water. The experimental protocol was approved by the Animal Welfare and Ethics Committee of the Military Medical University of the Army (AMUWEC20227002; Date**:** July 30, 2022). All procedures strictly adhered to established animal welfare guidelines, the National Institutes of Health (NIH) *Guide for the Care and Use of Laboratory Animals*, and the ARRIVE Guidelines (v2.0).

### Construction of a chronic intermittent hypoxia mouse model

In the control group (*n* = 37), the mice were housed in a normal air environment. For the CIH group (*n* = 62), the mice were placed in a hypoxic chamber with a system for controlling intermittent oxygen concentration (Pro-OX 100, Tawang Technology, Shanghai, China). The previous modeling regime was adjusted [28,29]. The analysis was conducted daily from 08:30 AM to 4:30 PM for approximately 8 weeks and involved alternating cycles of nitrogen and oxygen. The oxygen concentration was reduced from 21 to 6% over the first 30 s and maintained for at least 10 s, then increased back to 21% in the following 30 s to simulate a rate of 60 apneas/hour.

### Viral vectors and stereotactic surgery

In PVN (AP: −0.80 mm, ML: ±0.19 mm, DV: −4.8 mm):

AAV2/9 (rAAV-hSyn-mCherry-WPRE-Hgh polyA, 5.32E + 12 vg/mL, BrainVTA, Wuhan, China)
DIO-EGFP (rAAV-EF1a-DIO-EGFP-WPRE-Hgh polyA, 5.20E + 12 vg/mL, BrainVTA, Wuhan, China)
rAAV-EF1α-DIO-hM3D (Gq)-mCherry-WPREs (4.50E + 12 vg/mL, BrainVTA, Wuhan, China)
rAAV-EF1α-DIO-hM4D (Gi)-mCherry-WPREs (5.10E + 12 vg/mL, BrainVTA, Wuhan, China)
rAAV-EF1α-DIO-mCherry-WPREs-hGH polyA (5.35E + 12 vg/mL, BrainVTA, Wuhan, China)

In preBötC (AP: −6.80 mm, ML: ±1.35 mm, DV: −4.9 mm):

AAV2/Retro (rAAV-hSyn-EYFP-WPRE-Bgh pA 5.84E + 12 vg/mL, BrainVTA, Wuhan, China)
AAV2/9 (rAAV-hSyn-EYFP-WPRE-Hgh polya, 5.32E + 12 vg/mL, BrainVTA, Wuhan, China)
Retro-Cre (rAAV-hSyn-CRE-WPRE-Hgh polyA 5.45E + 12 vg/mL, BrainVTA, Wuhan, China) + CTB555 (BrainVTA, Wuhan, China)

The mice were fasted for 4–6 h preoperatively with free access to water. Turn on the gas anesthesia machine for small animals (RWD Life Science, Shenzhen, China; model: R500IQ) and the oxygen cylinder (≥99.5%). Isoflurane concentration (≥99.9%, RWD Life Science, Shenzhen, China; Model: R580-100ML) was set at 3% with oxygen flow at 0.5 L/min. The mice were then placed in an anesthesia induction chamber (RWD Life Science, Shenzhen, China; model: R510-22) filled with a gas mixture. Approximately 5 min after the loss of the righting reflex, adequately anesthetized mice were transferred to a brain stereotaxic instrument (RWD Life Science, Shenzhen, China) equipped with a mouse adapter. The nose was secured in a nasal mask, and anesthesia was maintained with 2% isoflurane with 0.5 L/min oxygen flow. Vital signs were assessed at 5-minute intervals. A direct-current heating pad beneath the mouse maintained body temperature at approximately 36 °C. Upon stabilization of respiration, absence of toe pinch and corneal reflexes, and muscle relaxation, the cranial hair was removed, and the scalp was disinfected. A midline incision was made in the skull skin, and the periosteum was cleared with a cotton swab to expose the bony landmarks. After leveling the head anteroposteriorly and laterally, viral injections were administered according to the coordinates of the PVN and the preBötC. To ensure adequate viral infection, wait 5 min after completing the injection at each site before removing the needle. Following stereotaxic viral injection, mice were transferred to a recovery chamber maintained at 32 °C. The surgical site was uniformly coated with 5% povidone-iodine ointment. Perioperative analgesia was administered *via* meloxicam (2 mg/kg, subcutaneous [SC], every 12 h) initiated preoperatively and continued for 72 h postoperatively. Enrofloxacin (5 mg/kg, SC, every 24 h) was administered as antibiotic prophylaxis for five days. The body weight and wound healing were monitored daily. According to the American Veterinary Medical Association (AVMA)guidelines, animals that lost more than 15% of their body weight or showed signs of distress were euthanized. Brain samples were sectioned and examined using confocal microscopy four weeks after viral transduction. See Table 1 for specific groupings.

**Table 1.** Stereotactic injection of viral vectors.

Experimental objective	Experimental group/operation (sample size)	Viral injection protocol
1. PVN→preBötC projection validation	i. Ipsilateral anterograde + retrograde labeling (n = 6)	PVN: AAV2/9-mCherry (100 nL) Ipsilateral preBötC: AAV2/Retro-EYFP (100 nL)
	ii. Targeting via Cre/LoxP system (n = 5)	Bilateral PVN: DIO-EGFP (80 ∼ 100 nL/side) Bilateral preBötC: Retro-Cre + CTB555 (5:1 mixture, 80 ∼ 100 nL/side)
2. PVN/preBötC → Phrenic nucleus	i. preBötC-originated labeling (n = 5)	preBötC: AAV2/9-EYFP (100 nL)
pathway validation	ii. PVN-originated labeling(n = 5)	PVN: AAV2/9-mCherry (100 nL)
3. Chemogenetic modulation experiment (*Viruses injected	i. Experimental group (Mice exposed to 4-week CIH) (hM3Dq/hM4Di: n = 8 each)	Bilateral PVN: DIO-hM3Dq or DIO-hM4Di (150 nL/side) Bilateral preBötC: Retro-Cre + CTB555 (100–150 nL/side)
post-4wk CIH + 4wk CIH maintenance*)	ii. Parallel control group (Mice exposed to 4-week CIH) (n = 16)	Bilateral PVN: DIO-mCherry (150 nL/side) Bilateral preBötC: Retro-Cre + CTB555 (5:1 mixture; total 100–150 nL/side)
	iii. Self-control group	Same cohort as Experimental Group (i)
4. Blood gas analysis experiment	CIH mice (n = 16; includes 8 mice from Exp. Group 3.i)	Bilateral PVN: DIO-hM3Dq (150 nL/side) Bilateral preBötC: Retro-Cre + CTB555 (100–150 nL/side)

### Chemogenetic stimulation and breathing measurements

#### Experimental grouping

The study involved three groups: (1) The experimental group: received DIO-hM3Dq or DIO-hM4Di virus and Retro-Cre virus, followed by intraperitoneal administration of the activator clozapine N-oxide (CNO) (i.e. DIO-hM3Dq/hM4Di + Retro-Cre + CNO). (2) The parallel-control group: received DIO-mCherry and Retro-Cre virus, followed by intraperitoneal administration of CNO(i.e. DIO-mCherry + Retro-Cre + CNO). (3) The self-control group: consisted of the same experimental mice, which were administered saline intraperitoneally on the second day after the CNO injection(i.e. DIO-hM3Dq/hM4Di + Retro-Cre + Saline).

#### Chemogenetic activation experiment

Respiratory indices in conscious and freely moving mice were assessed using the WBP (Buxco, St. Paul, MN, USA). The mice were allowed to acclimate in the test chamber for a minimum of 60 min before measurements. Both the parallel-control (mCherry) and experimental groups (hM3Dq) were administered an intraperitoneal injection of approximately 0.4 mL of CNO (0.1 mg/mL). Subsequently, respiratory parameters including respiratory frequency(RF), tidal volume (TV), and minute ventilation (MV) were continuously monitored for 2 h. MV and TV were normalized to body weight (g).

The effects usually lasted for about 4 h. Twenty-four hours after CNO injection, mice in the experimental group received an equal volume of saline for a self-control experiment, and respiratory parameters were monitored for 2 h.

#### Chemogenetic inhibition experiment

The study followed the same grouping requirements. This approach replicated the previously mentioned procedures. Respiratory parameters were recorded continuously for 2 h. Following the experiment, cardiac perfusion was conducted to aid in brain extraction, allowing for subsequent histological examination to verify the accuracy of the viral injection sites.

#### Blood collection from the abdominal aorta of mice

CIH mice (*n* = 16) received bilateral PVN injections of DIO-hM3Dq and bilateral preBötC injections of Retro-Cre + CTB555. Four weeks after injection, the mice were intraperitoneally administered either CNO (*n* = 8) or an equal volume of saline (*n* = 8). All mice were immobilized on a small-animal dissection table within 60 min of injection. Anesthesia was induced with 3% isoflurane, followed by a small abdominal incision to expose the internal organs, which were gently moved aside to reveal two adjacent blood vessels near the spine: a blue vein and a pulsating white artery. A segment of the arterial blood vessel was carefully isolated, and a 1 mL syringe was inserted parallel to the vessel to draw approximately 0.2 mL of arterial blood uniformly. After withdrawal, the needle tip was immediately inserted into a rubber plug to prevent air entry. Hemostatic forceps were applied to the artery for hemostasis, followed by cardiac perfusion to extract the brain for histological examination. After removing air bubbles, the collected arterial blood was promptly applied to an arterial blood gas analysis test strip. The test strip was inserted into a small-animal blood gas analyzer (i-STAT; Abbott; Chicago, IL) to obtain blood gas measurements.

#### Histology and immunoassay

After confirming deep anesthesia (absence of corneal and toe pinch reflexes), the mice were humanely euthanized through transcardial perfusion for subsequent tissue collection for histological experiments. The thoracic cavity of each mouse was quickly opened to expose the heart. Heparinized saline (37 °C) was perfused at 5–8 mL/min until the effluent ran clear, followed by ice-cold 4% paraformaldehyde (4 °C; in 0.1 M phosphate buffer, pH 7.4) at 10–15 mL/min. Perfusion continued until generalized limb rigidity developed. Brain tissues were extracted from mice after confirming death by dilated fixed pupils and absence of spontaneous respiration for more than 5 min, as well as a flat line on the ECG. Brain tissues were collected only from the regions necessary for this study. All procedures strictly adhered to the AVMA guidelines for Animal Euthanasia. The extracted brain tissues were fixed in 4% paraformaldehyde for 12 h, followed by dehydration in phosphate-buffered saline (PBS) containing 30% sucrose until the tissues sank. Post-dehydration, the brain tissues were embedded in optimal cutting temperature compound and sectioned into serial coronal slices from the olfactory bulb to the brain stem, each with a thickness of 40 µm. Sections were subsequently collected for fluorescence microscopy.

For both normoxic (*n* = 5) and CIH mice (*n* = 5), brain tissues underwent c-Fos staining. Sections covering the PVN and various other respiratory related nuclei were prepared. These sections were washed three times in PBS for 10 min each and permeabilized using an immunostaining permeabilization solution for 1 h, followed by another round of PBS washes. Subsequent to blocking with an immunostaining solution at room temperature for 2 h, the sections were exposed to the primary antibody (c-Fos primary antibody, 1:400; #2250 Cell Signaling Technology, Danvers, MA) overnight at 4 °C. Following additional PBS washes, the sections were treated with the secondary antibody (1:500; A-21206 Invitrogen, Waltham, MA) at room temperature for 2 h, washed again in PBS, and finally sealed with a DAPI-containing sealer. The samples were analyzed using a confocal microscope (Nikon AX, Tokyo, Japan). The quantity of c-Fos-positive neurons in the relevant nuclei was manually enumerated, the total area of each respiratory-associated nucleus was gauged in each field of view; and the number of c-Fos-positive neurons in each respiratory-associated nucleus was standardized to neurons/mm^2^.

To elucidate c-Fos expression in the preBötC and the PVN neurons, we collected six consecutive coronal sections from each of the two brain regions in DIO-hM3Dq and Retro-Cre injected CIH mice. The mice were intraperitoneally administered CNO or saline. After 90 min, they were subjected to cardiac perfusion for brain extraction. Furthermore, immunoreactivity assays for c-Fos in the brain tissue were conducted. The brain sections were incubated with primary antibodies against c-Fos (#2250; Cell Signaling Technology Danvers, MA) (dilution 1:400) at 4 °C for 24–48 h. Sections were washed with PBS. Secondary antibodies, donkey anti-rabbit AF488 (A-21206; Invitrogen, Waltham, MA), were prepared at a dilution of 1:500 and applied to the brain slices for 2-hour incubation at room temperature. After subsequent PBS washes, slices were sealed with a DAPI-containing sealer. Confocal microscopy (Zeiss, Oberkochen, Germany) was used for visualization and c-Fos-positive neurons in the preBötC and the PVN were counted manually.

#### Statistical analysis

Statistical analyses were performed using GraphPad Prism 9.5.1 software. Experimental results were expressed as mean ± standard error of the mean (SEM). Data comparisons between groups were conducted using paired or unpaired t-tests or one-way ANOVA as appropriate. Two-way analysis of variance (ANOVA) and repeated measures ANOVA were employed to analyze differences between and within groups. A *p* value < 0.05 was considered statistically significant.

## Result

### Increased expression of c-fos in CIH mice

**Figure 1.**
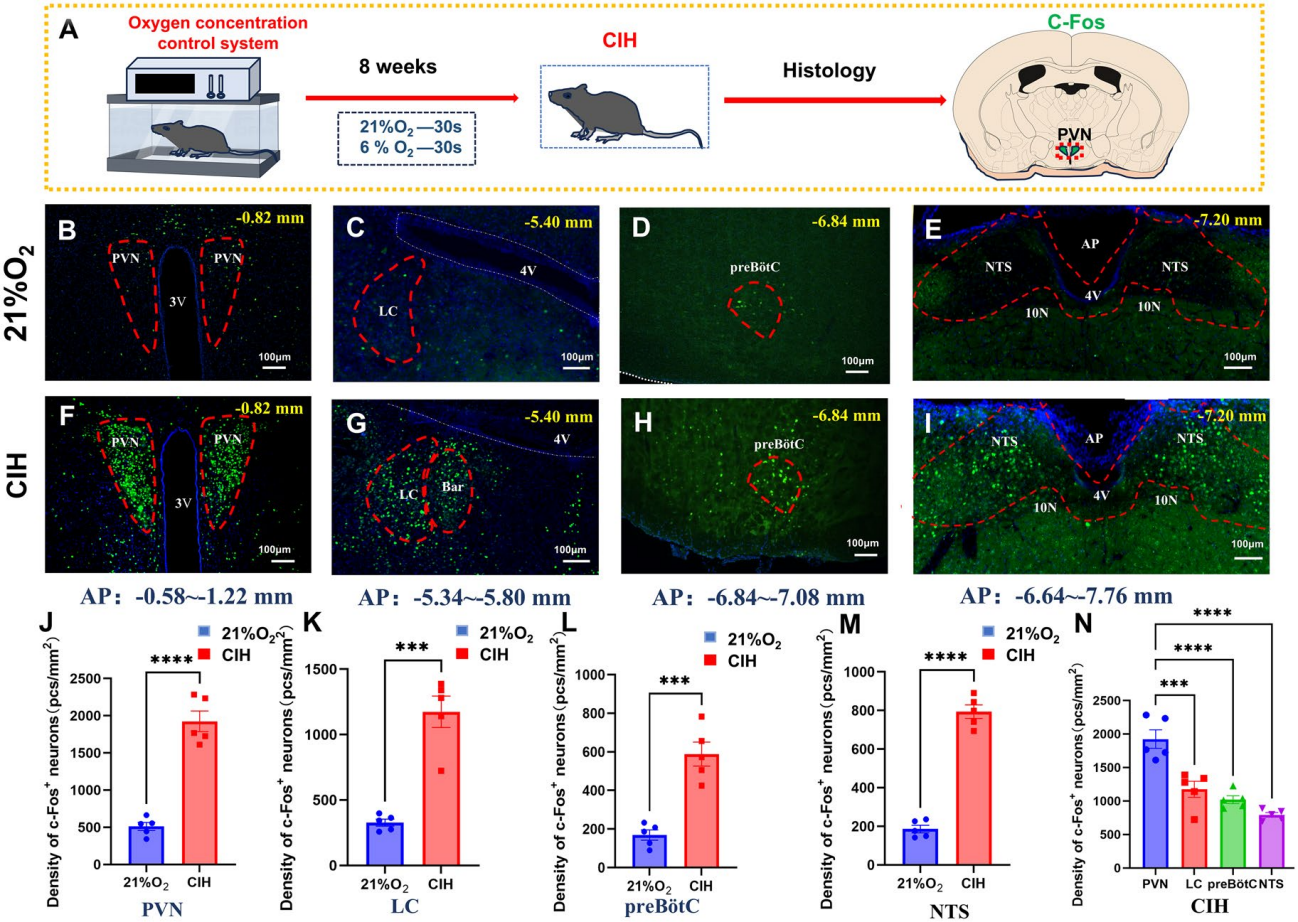
Increased expression of c-Fos in CIH mice. A: Schematic diagram of the experimental protocol: Mice were placed in an oxygen concentration control system for 8 weeks to establish a chronic intermittent hypoxia(CIH) mouse model. C-Fos staining was performed in brain regions including the paraventricular hypothalamic nucleus (PVN). B–I. Micrographs showing c-Fos-positive neurons (green) in four brain regions: PVN, Locus coeruleus (LC), pre-Bötzinger complex (preBötC), and nucleus tractus solitarius (NTS) from the 21% O2 group (*n* = 5) and CIH group (*n* = 5). J–N. Quantitative analyses comparing the density of c-Fos-positive neurons (normalized as neurons/mm2) per brain region between groups. There was a statistically significant increase in neuronal density was observed in all CIH-group regions compared to controls (21% O2 group) (****p < 0.0001, unpaired t-test), with the highest density in the PVN (****p < 0.0001, one-way ANOVA). Annotations: ‘4V’ denotes the 4th ventricle; ‘3V’ the 3rd ventricle; ‘AP’ the area postrema; ‘10N’ the dorsal motor nucleus of the vagus; ‘CC’ the central canal.

Brain slices from the PVN, Locus coeruleus (LC), NTS, and preBötC of both normoxic and CIH mice were examined, and c-Fos-positive neurons in these regions were manually counted (Figure 1A–I). Discrepancies were assessed by enumerating the number (density) of c-Fos-positive neurons per unit area. CIH mice displayed elevated mean densities of c-Fos-positive neurons in all respiratory nucleus brain slices (Figure 1J–M). (PVN: 1925.0 ± 139.1 in CIH *vs*. 510.9 ± 54.32 in 21% O_2_, *t(8)* =9.470, *p* < 0.0001; LC: 1174.0 ± 120.2 in CIH *vs.* 327.5 ± 26.53 in 21% O_2_, *t(8)*=6.883, *p* = 0.0001; NTS:793.5 ± 35.06 in CIH *vs.* 186.0 ± 19.37 in 21% O_2_, *t(8)* = 15.17, *p <* 0.0001; preBötC: 588.4 ± 61.92 in CIH *vs.* 168.7 ± 26.38 in 21% O_2_, *t(8)*=6.236, *p =* 0.0002; Unit: neurons/mm^2^). Moreover, the PVN demonstrated a greater density of c-Fos-positive neurons compared to other brain regions (Figure 1N, *p* < 0.0001).

### Projection relationship analysis between the PVN and the preBötC

**Figure 2.**
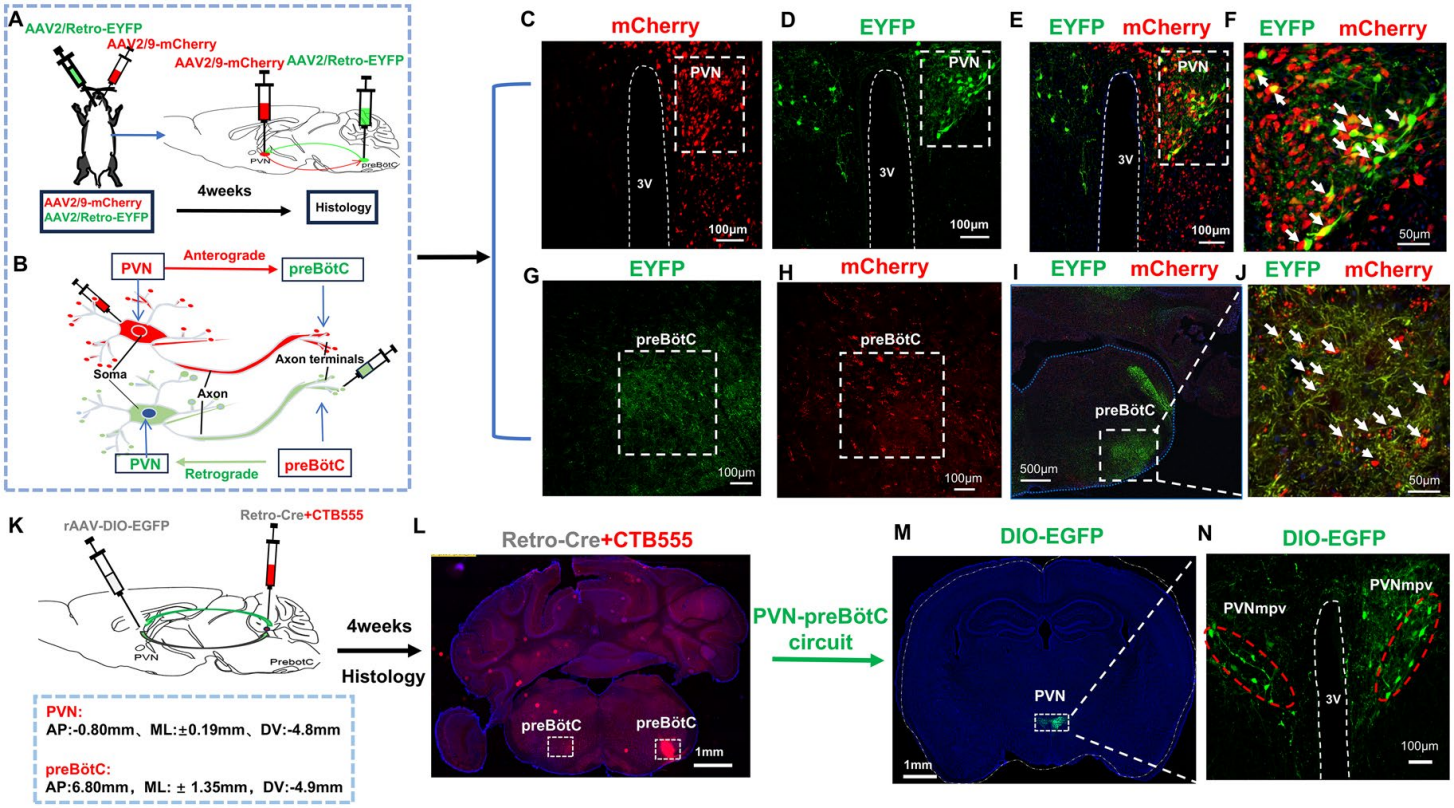
Analysis of projection relationship between PVN and preBötC. A. Schematic diagram of the experimental approach: An anterograde non-transsynaptic tracing virus (AAV2/9-mCherry) was injected into the paraventricular hypothalamic nucleus (PVN), while a retrograde non-transsynaptic tracing virus (AAV2/R-EYFP) was injected into the ipsilateral pre-Bötzinger complex (preBötC). Brains were collected by cardiac perfusion after four weeks of viral transduction (*n* = 6). B. Schematic illustration of non-synaptic viral tracing: Anterograde tracing: projection from neuronal somata to nerve fiber terminals; retrograde tracing: projection from nerve fiber terminals to neuronal somata. C. Confocal micrographs (single channe): Anterogradely labeled red neuronal somata in the PVN; D. Single channel: Green retrogradely labeled neuronal somata in the PVN projecting from the preBötC. E. Co-localization in the PVN: Red anterogradely labeled neuronal somata with green retrogradely labeled neuronal somata from the preBötC; F. Magnified view of co-labeled neuronal somata in PVN (white arrows). G. Single channel: Retrograde green nerve fibers in the preBötC. H. Single channel: Red anterograde fibers from the PVN to the preBötC. I-J. Co-localization of retrograde green fibers (preBötC) and anterograde red fibers (PVN) in preBötC (white arrows). K. Experimental schematic: rAAV-DIO-EGFP injected into bilateral PVN and Retro-Cre + CTB555 (5:1 mixture) into bilateral preBötC. Brains collected by cardiac perfusion after 4 weeks (*n* = 5). L-M. Confocal micrographs: Bilateral preBötC injection sites and DIO-EGFP expression in PVN via the PVN-preBötC circuit. N. Magnified view of infected neurons in bilateral PVN: EGFP-positive neurons (green) concentrated in the ventrolateral PVN, predominantly within the PVN mpv subregion.

Distinct tracing of viruses was utilized to establish a direct projection relationship between the PVN and the preBötC. An anterograde non-transsynaptic tracing virus (AAV2/9-mCherry) was injected into the PVN, while a retrograde non-transsynaptic tracing virus (AAV2/R-EYFP) was administered to the ipsilateral preBötC. After four weeks, the brains were harvested *via* cardiac perfusion (Figure 2A,B). Confocal microscopic examination of brain slices revealed co-labeling (yellow) in the PVN region, where the retrogradely labeled green neuronal somata (from preBötC) overlapped with the anterogradely labeled red neuronal somata (injected into the PVN) (Figure 2C–F). Furthermore, in the preBötC, red anterograde and green retrograde-labeled nerve fibers were co-labeled (Figure 2G–J), confirming a direct projection relationship between the PVN and the preBötC. Subsequent injection of DIO-EGFP in the PVN and Retro-Cre in the preBötC facilitated the identification of EGFP-positive neurons in the PVN (Figure 2K–N). These EGFP-positive neurons (green) were predominantly concentrated in the ventral-lateral region of the PVN, particularly in the paraventricular hypothalamic nucleus, medial parvicellular part(PVNmpv) (Figure 2N), representing 57.49% ± 3.47% of all EGFP-positive neurons in the PVN.

### Connection between the preBötC and the spinal phrenic nucleus

**Figure 3.**
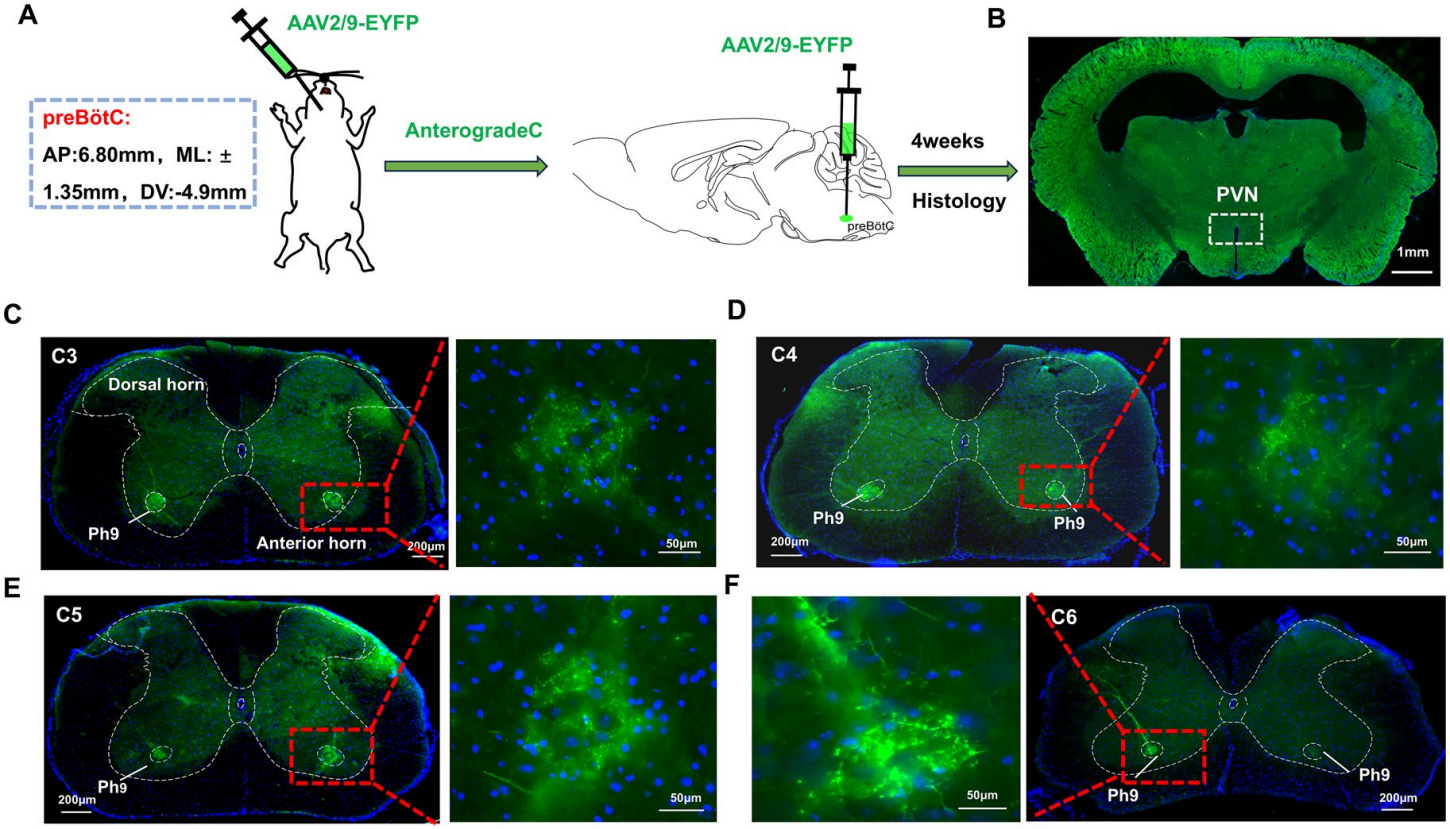
Connection between the preBötC and the spinal phrenic nucleus. A. Schematic diagram of the experimental setup: An anterograde non-transsynaptic virus (AAV2/9-EYFP) was injected into the pre-Bötzinger complex (preBötC). Four weeks post-infection, the paraventricular hypothalamic nucleus (PVN) and the spinal phrenic nucleus were examined histologically (*n* = 5). B. Micrographs show no obvious anterogradely labeled EYFP-positive nerve fibers in the PVN. C–F. Photomicrographs reveal anterogradely labeled EYFP-positive nerve fibers in the phrenic motoneurons of lamina 9 (Ph9) region at C3–C6 spinal cord levels. Anatomical details of the C3–C5 Ph9 region: Left panels: Ph9 boundaries delineated by red dashed lines; Right panels: Magnified views of nerve fibers within Ph9(C, D, E). C6 Ph9 region: Left: Magnified nerve fibers in Ph9; Right: Ph9 contour outlined with red dashed line(F).

**Figure 4.**
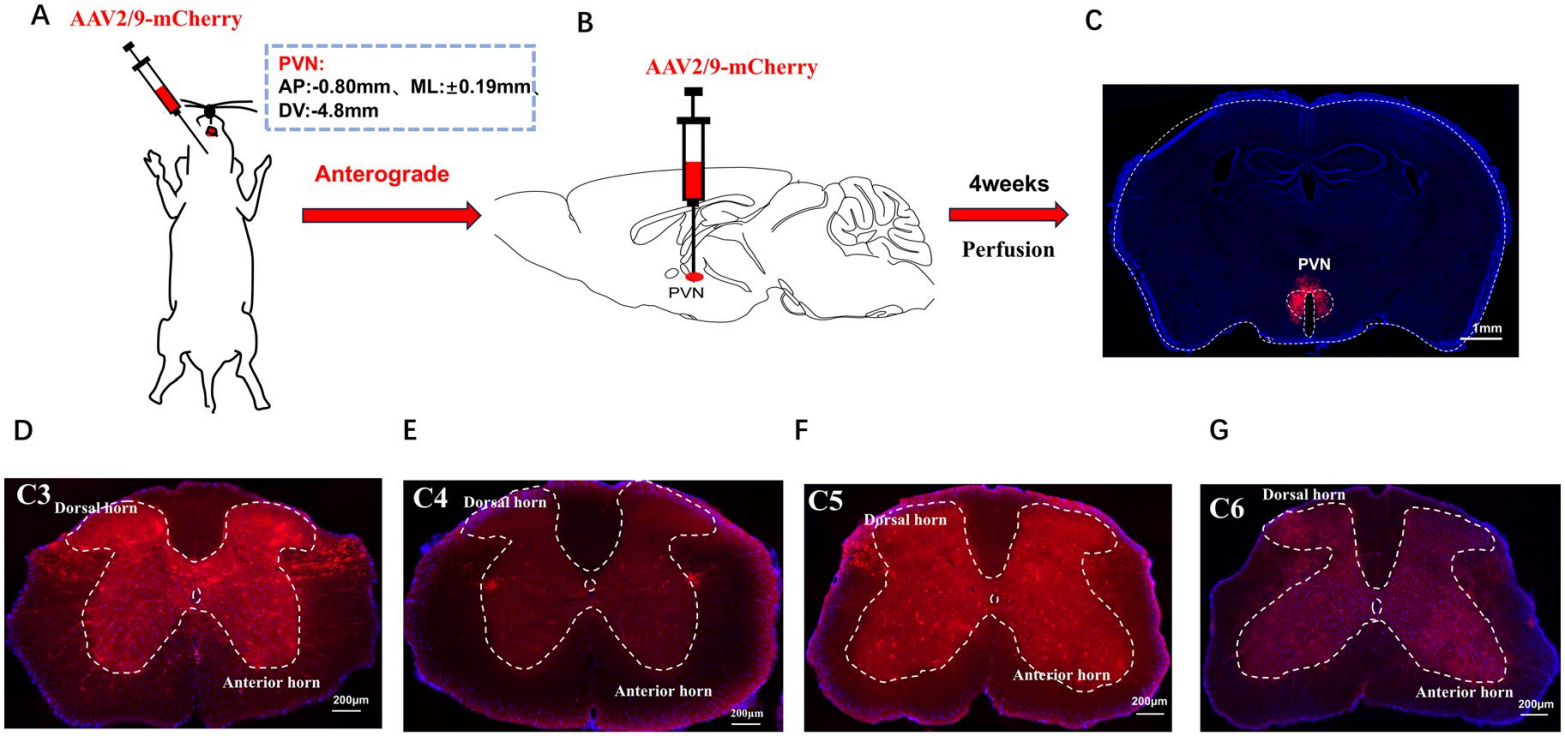
Projections of the PVN to the spinal phrenic nucleus. A-B. Schematic diagram of the experimental setup: An anterograde non-transsynaptic virus (AAV2/9-mCherry) was injected into the paraventricular hypothalamic nucleus (PVN). Four weeks post-infection, histological examination of the spinal phrenic nucleus was performed (*n* = 5). C. Photomicrographs show: verification of PVN injection sites. D-G. Photomicrographs show no detectable red nerve fibers in the phrenic motoneurons of lamina 9 (Ph9) region of the C3∼C6 spinal cord.

To investigate whether the preBötC directly connected to the spinal phrenic nucleus, researchers injected an anterograde non-transsynaptic virus (AAV2/9-EYFP) into the preBötC (Figure 3A). Four weeks after the infection, they observed anterograde green nerve fibers in the spinal phrenic nucleus, primarily in the phrenic motoneurons of lamina 9 (Ph9) region (Figure 3C–F). However, no EYFP-positive(green) nerve fibers were found in the PVN (Figure 3B). In contrast, when AAV2/9-mCherry was injected into the PVN (Figure 4A–C), researchers did not detect significant anterograde red nerve fibers in the spinal phrenic nucleus (Figure 4D–G).

### Effect of chemogenetic activation of PVN neurons on their basal ventilation

**Figure 5.**
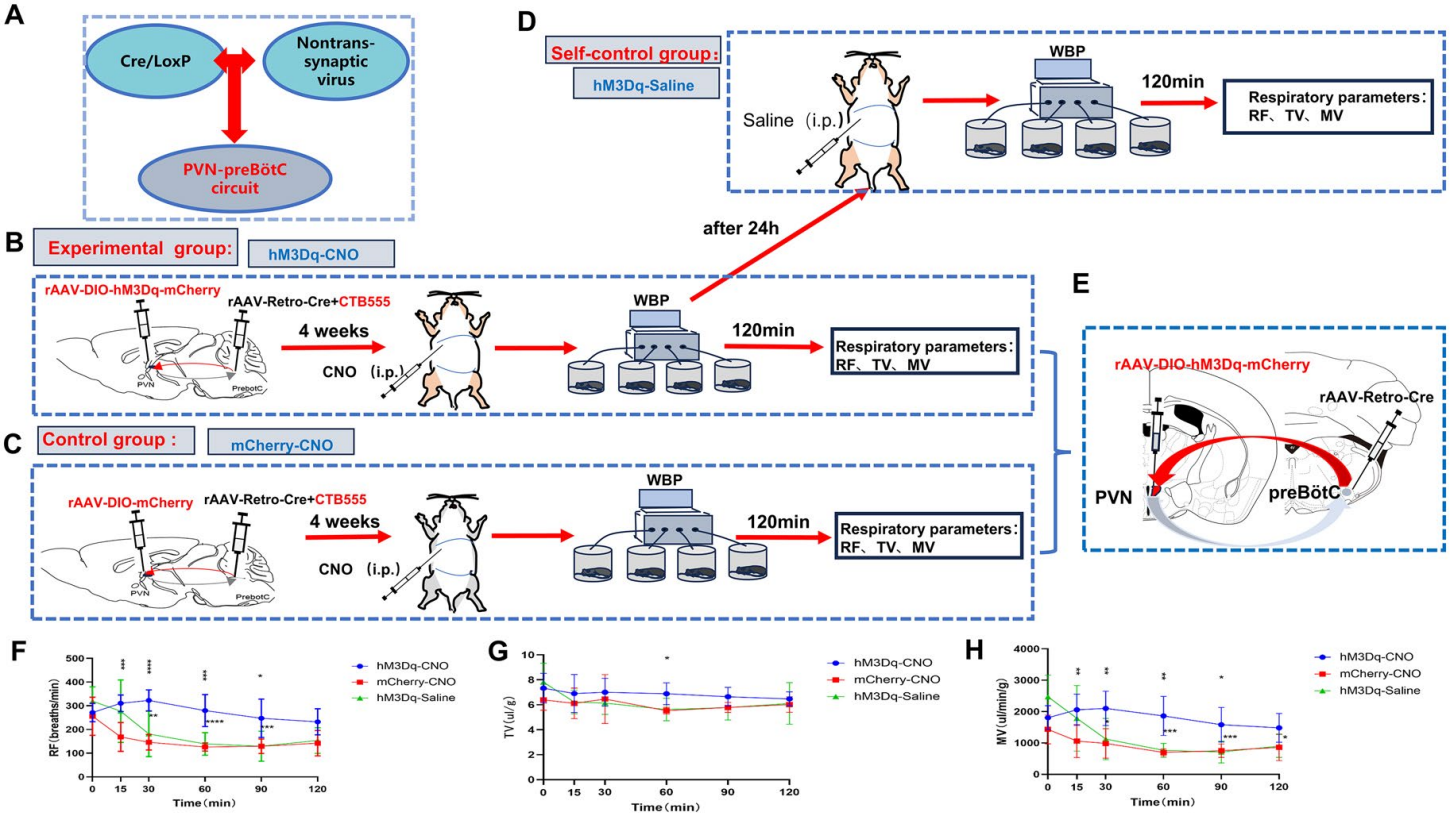
Impact of chemogenetic activation of PVN neurons on respiratory parameters in CIH mice. A. Schematic illustration of the Cre/LoxP expression strategy combined with non-transsynaptic viral tracing to validate the PVN-preBötC neural circuit. B-C. Experimental protocol: Chronic intermittent hypoxia(CIH) mice received bilateral injections of DIO-hM3Dq (*n* = 8) or DIO-mCherry (*n* = 8) into the paraventricular hypothalamic nucleus (PVN), and Retro-Cre + CTB555 (5:1 mixture) into the pre-Bötzinger complex (preBötC). Four weeks post-injection, mice were administered clozapine-N-oxide (CNO) followed by whole-body plethysmography (WBP) to measure respiratory frequency (RF), tidal volume (TV), and minute ventilation (MV). D. After 24 hours, experimental mice received saline injections followed by repeat respiratory testing. E. Schematic of bilateral PVN and preBötC virus injections. F-H. Chemogenetic stimulation effects on respiratory parameters: CNO activation in hM3Dq-expressing mice significantly increased RF and MV , without TV changes. No notable alterations in respiratory parameters were observed in the parallel-control (mCherry) and self-control (Saline) groups. (Statistical significance indicated as *p < 0.05, **p < 0.005, ***p < 0.001, ****p < 0.0001, #*p* < 0.05, ##*p* < 0.005, ###*p* < 0.001, #### *p* < 0.0001; was analyzed using two-way and repeated-measures ANOVA).

Four weeks after DIO-hM3Dq/mCherry and Retro-Cre virus injection, respiratory parameters, such as RF, TV, and MV, were measured in both the experimental and control groups using WBP **(**Figure 5A–E). In the experimental group, RF and MV increased following intraperitoneal administration of CNO, while TV remained relatively stable. RF showed a gradual increase post-CNO injection, reaching a peak at 30 min (323 ± 16 *vs.* 146 ± 12 *vs.* 181 ± 34 breaths/min, hM3Dq-CNO *vs.* mCherry-CNO *vs.* hM3Dq-Saline, F (1.228, 8.599) = 28.79, *p* = 0.0004), and returning to baseline levels by 90 min (323 ± 16 *vs.* 247 ± 28, 30 min *vs.* 90 min, F (2.463, 17.24) = 10.78, *p* = 0.0005). MV also exhibited a gradual increase post-injection, peaking at 30 min (2102 ± 194 *vs.* 986 ± 167 *vs.* 1123 ± 233 UL/min/G, hM3Dq-CNO *vs.* mCherry-CNO *vs.* hM3Dq-Saline, F (1.751,12.26) = 15.78, *p* = 0.0005), and returning to baseline levels around 90 min (2102 ± 194 *vs.* 1582 ± 194, 30 min *vs.* 90 min, F (2.625, 18.37) = 10.78, *p* = 0.0002). In contrast, the parallel control group did not show significant changes in respiratory parameters after CNO injection. Similarly, there were no notable changes in respiratory parameters in the self-control group following saline injection (Figure 5F–H). These results suggest that the activation of PVN neurons significantly enhanced basal MV by increasing RF, without affecting TV.

**Figure 6.**
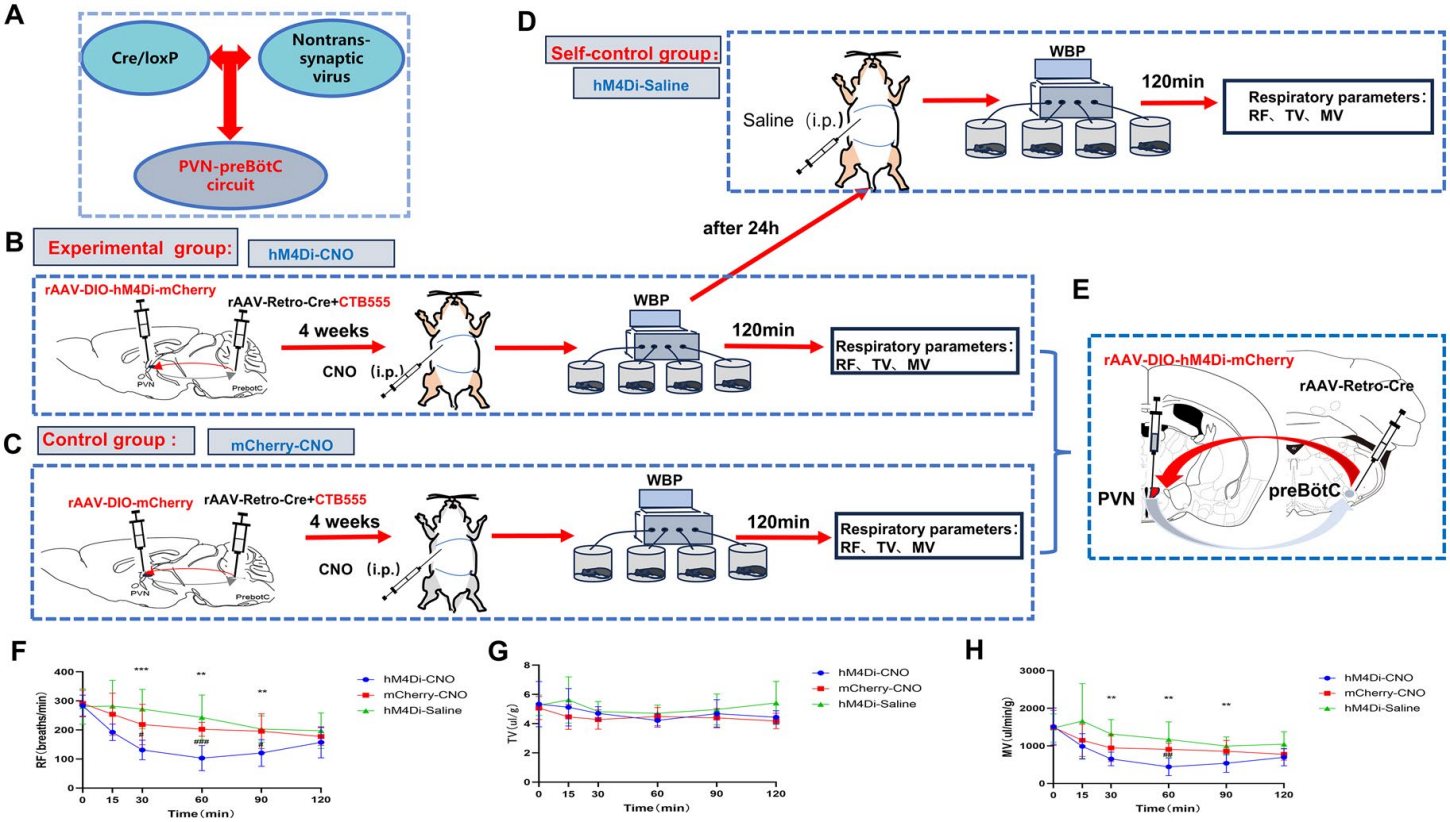
Effects of chemogenetic inhibition of PVN neurons on basal ventilation in CIH mice. A. Schematic illustration of the Cre/LoxP expression strategy combined with non-transsynaptic viral tracing to validate the PVN-preBötC neural circuit. B-C. Experimental protocol: Chronic intermittent hypoxia(CIH) mice received bilateral paraventricular hypothalamic nucleus (PVN) injections of DIO-hM4Di (*n* = 8) or DIO-mCherry (*n* = 8), and pre-Bötzinger complex (preBötC) injections of Retro-Cre + CTB555 (5:1 mixture). After 4-week viral expression, mice underwent intraperitoneal clozapine-N-oxide (CNO) administration followed by whole-body plethysmography (WBP) to measure respiratory frequency (RF), tidal volume (TV), and minute ventilation (MV). D. At 24 hours post-CNO, experimental mice received saline injections with repeat WBP testing. E. Schematic of bilateral PVN and preBötC virus injections. F-H. Chemogenetic inhibition effects: CNO activation in hM4Di-expressing mice significantly decreased RF and MV, without TV changes. No alterations occurred in Parallel-control (mCherry) and self-control (Saline) groups. (Statistical significance indicated as *p < 0.05, **p < 0.005, ***p < 0.001, ****p < 0.0001; #*p* < 0.05, ##*p* < 0.005, ###*p* < 0.001; two-way and repeated-measures ANOVA).

Four weeks after DIO-hM4Di/mCherry and Retro-Cre virus injection, respiratory parameters, including RF, TV, and MV were measured in both the experimental and control groups using WBP (Figure 6A–E). In the experimental group, RF and MV decreased following intraperitoneal administration of CNO, while TV remained relatively stable. RF decreased gradually after CNO injection, reaching its lowest point at 60 min (103 ± 15 *vs.* 203 ± 8 *vs.* 244 ± 27 breaths/min, hM4Di-CNO *vs.* mCherry-CNO *vs.* hM4Di-Saline, F (2, 21) = 9.548, *p* = 0.0011), and then gradually increased to nearly baseline levels by 120 min (103 ± 15 *vs.* 158 ± 19, 60 min *vs.* 120 min, F (3.478, 73.05) = 24.75, *p* < 0.0001). MV similarly decreased gradually, reaching a trough at 60 min (444 ± 83 *vs.* 909 ± 58 *vs.* 1170 ± 165 UL/min/G, hM4Di-CNO *vs.* mCherry-CNO *vs.* hM4Di-Saline, F (2, 21) = 6.485, *p* = 0.0064), and returned to a level close to baseline after 120 min (444 ± 83 *vs.* 695 ± 80, 60 min *vs.* 120 min, F (2.819, 59.20) = 17.65, *p* < 0.0001). In contrast, there was no significant change in respiratory indices after CNO injection in the parallel control group. Similarly, no significant changes in respiratory parameters were observed in the self-control group following saline injection (Figure 6F–H). These results suggest that the inhibition of PVN neurons significantly reduces basal MV by decreasing RF, with no substantial effect on TV.

### Effect of chemogenetic activation on blood gas in mice

**Figure 7.**
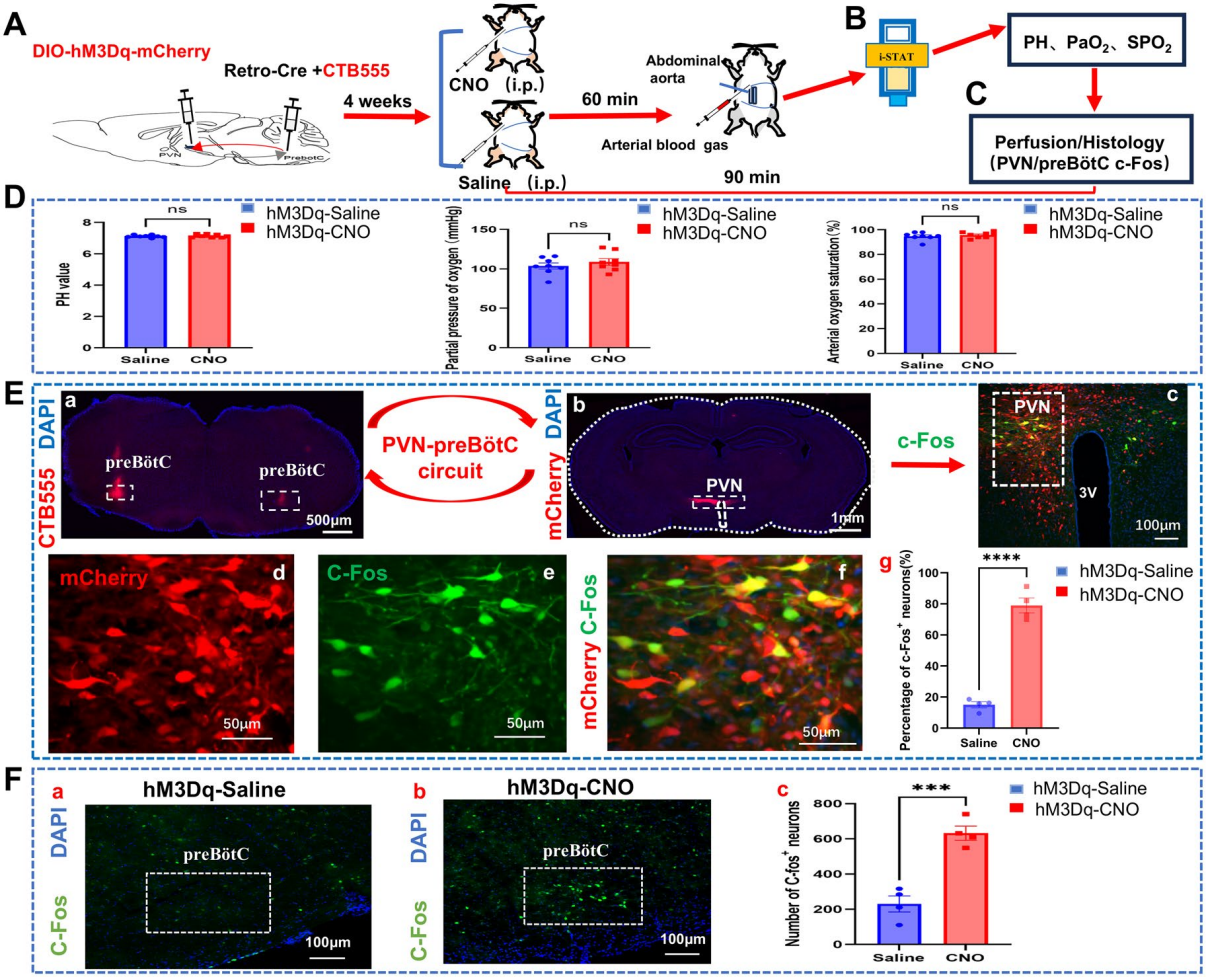
Impact of chemogenetic activation of PVN neurons on blood gas levels and on c-Fos expression in preBötC of CIH mice. A. Experimental protocol: Chronic intermittent hypoxia (CIH) mice received bilateral paraventricular hypothalamic nucleus (PVN) injections of DIO-hM3Dq and pre-Bötzinger complex (preBötC) injections of Retro-Cre/CTB555 (5:1 mixture). Four weeks post-injection, mice received intraperitoneal saline or clozapine-N-oxide (CNO) (*n* = 8/group). B. Arterial blood gas analysis 60 minutes post-injection. C. 90 minutes post-injection, c-Fos staining and quantification on serial coronal sections of PVN and preBötC. D. No significant differences in arterial blood pH, PaO2, or oxygen saturation between hM3Dq-Saline (control, *n* = 8) and hM3Dq-CNO (experimental, n = 8) groups (p &gt; 0.05, unpaired t-test). E.a-b: Confocal micrographs showing bilateral preBötC injection sites and DIO-mCherry expression in PVN via PVN-preBötC circuit. c: c-Fos+/mCherry + co-localization in PVN of CNO-activated mice. d: mCherry + neurons in PVN (single channel). e: c-Fos + neurons in PVN (single channel). f: Magnified view of co-labeled neurons. g: Higher proportion of c-Fos+/mCherry + co-labeled neurons in hM3Dq-CNO group (n = 4) vs. hM3Dq-Saline controls (*n* = 4) (****p < 0.0001, unpaired t-test). F.a-b: Confocal micrographs showing increased c-Fos + neurons in preBötC of hM3Dq-CNO group (*n* = 4) vs. hM3Dq-Saline (*n* = 4). c: Quantification of c-Fos + neurons in preBötC (***p = 0.0006, unpaired t-test analysis).

To assess the impact of activating PVN neurons on blood gas parameters in CIH mice, arterial blood gas analysis was conducted four weeks post-hM3Dq virus injection. The mice received intraperitoneal injections of either saline (control group, *n* = 8) or CNO (experimental group, *n* = 8) (Figure 7A,B). The findings revealed no significant variances between the control and experimental groups regarding pH (7.122 ± 0.029 *vs.* 7.142 ± 0.035, Saline *vs.* CNO, *t* (14) = 0.4238, *p* = 0.6782), oxygen partial pressure (103.6 ± 3.8 *vs.* 108.8 ± 4.2 mmHg, Saline *vs.* CNO, *t* (14) = 0.9066, *p* = 0.3799), blood oxygen saturation (94.75 ± 1.11% *vs.* 95.75 ± 0.82%, Saline *vs.* CNO, *t* (14) = 0.7234, *p* = 0.4813), or other blood gas metrics (Figure 7D).

### Effect of PVN neuron activation on preBötC c-fos expression in mice

To assess the PVN neuron activity following CNO activation and determine if PVN neuron stimulation enhances baseline ventilation *via* preBötC neuron expression, we quantified c-Fos expression in the PVN and preBötC post-CNO stimulation. CIH mice were administered either saline (control group, *n* = 4) or CNO (experimental group, *n* = 4) following bilateral injection of DIO-hM3Dq in the PVN and Retro-Cre virus in the preBötC region. Subsequently, c-Fos staining was conducted on consecutive coronal brain sections of the PVN and preBötC (Figure 7A,C,Ea–f,Fa–b), and the number of c-Fos-positive neurons was quantified. In comparison to the control group, the experimental group exhibited a significantly higher proportion of c-Fos-positive neurons co-labeled with mCherry-positive neurons in the PVN (78.99% ± 4.814% *vs.* 15% ± 1.949%, CNO *vs.* Saline, *t (6)* = 12.32, *p* < 0.0001) (Figure 7Eg), while also showing a notable increase in c-Fos–positive neurons in the preBötC region (632 ± 40 *vs.* 230 ± 46, CNO *vs.* Saline, t (6) = 6.592, *p =* 0.0006) (Figure 7Fc).

## Discussion

Among the primary pathological factors of OSAHS, CIH is the most characteristic *in vivo* condition, posing multiple serious health risks that necessitate effective treatment [3]. This study demonstrated that, under CIH conditions, the expression of respiration-related nuclei was enhanced, as evidenced by c-Fos staining, with the PVN showing particular sensitivity to CIH stimulation. Neuroanatomical evidence for a direct projection relationship between the PVN and preBötC was obtained using viral tracing techniques. Furthermore, chemogenetic experiments in conscious CIH mice confirmed that the stimulation of PVN neurons could alter basal ventilation by modulating the RF without affecting arterial blood gas. These changes may be associated with the PVN-preBötC circuit, which is involved in the regulation of respiration, corroborating our previously proposed scientific hypothesis.

The preBötC neuron plays a central role in the respiratory central pattern generator (rCPG) [10], Previous studies have emphasized the PVN’s significant role in the respiratory neural network, but evidence of direct synaptic connections between the PVN and respiratory network neurons remains elusive. leading to skepticism about the PVN’s role in respiratory regulation. In this study, an anterograde nontranssynaptic virus (AAV2/9) was injected into the PVN, and a retrograde nontranssynaptic virus (AAV2/R) was injected into the ipsilateral preBötC. As these viruses are non-trans-synaptic, they label direct projection paths of specific neurons; targeting only one level of the region above and below. Co-labeled neurons or nerve fibers were observed in both the PVN and preBötC, demonstrating a direct projection relationship between them. The PVN acts as the upstream input nuclei to the preBötC, while the preBötC acts as the downstream output nuclei of the PVN. Since viruses carrying the DIO element are expressed only in Cre-expressing animals, to further validate the direct projection relationship between the PVN and preBötC, the Cre/LoxP gene expression strategy was used in combination with tracer viruses. By injecting DIO-EGFP into the PVN and Retro-Cre virus into the preBötC, the PVN-preBötC circuit was defined. The presence of an EGFP-positive neuronal soma in the PVN after virus infection verified the existence of a direct PVN-preBötC circuit. Since the virus are non-transsynaptic, it also reaffirms the direct synaptic connection between the two nuclei. In addition, most of the EGFP-positive neurons in the PVN are concentrated in the ventral-lateral region, specifically the PVNmpv region, which contains most descending preautonomic neurons projecting downstream to the brainstem and/or spinal cord [30].

Previous studies using CTB virus injections into the diaphragm identified retrograde-labeled neurons in the PVN [22], suggesting a projection relationship between the PVN and the spinal phrenic nucleus. However, evidence for anterograde tracing is lacking. Studies using retrograde tracer viruses such as CTB or pseudorabies virus (PRV) in the diaphragm and intercostal muscles have identified retrogradely labeled phrenic and intercostal motor neurons in the spinal cord [31]. These two neurons are the main spinal respiratory motor neurons and provide an anatomical basis for studying spinal respiratory motor neural networks. However, a morphological connection between the spinal respiratory motor neural network and the upstream respiratory center output nuclei is lacking.

When tracing the spinal cord region of the PVN, anterograde non-trans-synaptic projection did not show clear projection fibers in the spinal phrenic nucleus region. This absence may be attributed to the limited transmission range of non-trans-synaptic viruses and the considerable distance between the PVN and the spinal phrenic nucleus. Future studies should consider utilizing anterograde trans-monosynaptic viruses or other tracers for validation. Alternatively, a direct projection between the PVN and the spinal phrenic nucleus may not exist. In contrast, the anterograde non-transsynaptic virus injected into the preBötC revealed fiber projections in the cervical spinal cord after four weeks. The majority of nerve fibers were concentrated in the Ph9 region, where phrenic motor neurons are predominantly distributed in the cervical medulla, confirming the direct projection relationship between the preBötC and the spinal phrenic nucleus [31]. However, no evident green nerve fibers were observed in the PVN. Combining these results with previous viral tracer findings, it is evident that the PVN serves as a direct input region to the preBötC, while the phrenic nucleus acts as a direct output region of the preBötC. These findings provide an anatomical foundation for further exploration of the entire respiratory motor neural network.

Studies have shown that the PVN can enhance the projection of catecholinergic neurons to the NTS under acute hypoxia, thus contributing to respiration regulation [24,32–34]. However, the PVN’s role in respiratory regulation within the CIH environment remains unclear [27,35]. In this study c-Fos staining in CIH mice revealed a notably higher density of c-Fos-positive neurons in the PVN than in other respiratory-related nuclei, indicating an increased sensitivity of the PVN to CIH stimulation. These findings suggest the PVN as a potential therapeutic target for diseases associated with CIH. Previous studies have shown that electrical stimulation of the PVN in anesthetized rabbits can elevate the respiratory rate [36]. Similarly, glutamate injection into the PVN increased the myoelectric activity of the diaphragm in anesthetized rats [20]. However, these experiments were conducted on anesthetized animals. In contrast, this study utilized a CIH mouse model for chemogenetic experiments that incorporated WBP to evaluate respiratory parameters. WBP, a noninvasive pulmonary function test, though less precise than invasive methods, allows the assessment of conscious, freely moving animals. Its non-invasive nature reduces animal discomfort and permits repeated testing of the same subjects.

In this study, Retro-Cre viruses were injected into the preBötC, and DIO-hM3Dq or DIO-hM4Di viruses were administered in the PVN to establish upstream and downstream connectivity of the PVN-preBötC, respectively, to test respiratory function after activating or inhibiting the PVN, thereby verifying that the PVN regulates respiration in the CIH environment and can only be specifically expressed *via* the PVN-preBötC circuit (Figures 5A,E and 6A,E). This study comprehensively examined the impact of the PVN on respiratory regulation in CIH by investigating both activation and inhibition. Upon PVN neuron activation or inhibition, basal ventilation in mice varied with changes in RF, while TV remained relatively constant. Given that MV = RF × TV, it was observed that stimulation of PVN neurons in a CIH environment altered RF, but not TV, thereby increasing basal MV through RF modifications. This pattern aligns with the human respiratory behavior, where the RF is primarily adjusted to maintain adequate resting ventilation [37]. RF and MV in the parallel-control and self-control groups decreased over time, likely due to the mild suppression of respiratory function in mice confined in a limited closed space for extended durations.

Additionally, arterial blood gas analysis in mice activated with hM3Dq revealed that PVN neuron stimulation had no significant impact on the pH, oxygen partial pressure, or oxygen saturation, among other blood gas parameters. This suggests that while PVN activation alters ventilation, it does not influence oxygenation or the acid-base balance. Nonetheless, normal blood gas values do not rule out abnormalities in respiratory physiology, which may stem from intricate compensatory mechanisms achieved through dynamic adjustments of ventilation/perfusion ratios (V/Q), chemoreflex modulation, and acid-base buffering. Subsequent c-Fos immunostaining studies in DIO-hM3Dq mice showed a notably higher percentage of c-Fos-positive neurons in the PVN in the experimental group compared to the control group. This observation indicates that the significant increase in PVN neuronal activity following CNO activation underscores the efficacy of the designer receptors exclusively activated by designer drugs (DREADD) system in this study [25,38]. Additionally, the number of c-Fos-positive neurons in the preBötC region was markedly elevated in the experimental group relative to the control group. Retro-Cre was administered into the preBötC and Cre DREADDs were released in the PVN, confirming the PVN-preBötC circuit. The activation of PVN neurons was deduced to transmit directly *via* the PVN-preBötC circuit, enhancing their activity in response to inputs from the preBötC.

The PVN is a highly integrated nucleus that regulates neuroendocrine and cardiorespiratory functions. Increased PVN expression in CIH alters ventilation function, potentially through cumulative effects. Respiration is primarily regulated *via* the PVN-preBötC circuit, which may also be influenced by behavioral arousal outcomes such as stress and anxiety. This effect could involve mechanisms like activation of the hypothalamic-pituitary-adrenal (HPA) axis or the hypothalamic-pituitary-thyroid (HPT) axis, leading to hormone release that modulates body functions. Some studies have also suggested that RF and TV may be mobilized differently when exposed to various stressors [39]. Additionally, under hypoxic conditions, PVN neurons can directly project to the NTS *via* autonomic pathways, activating neuronal responses within the NTS without relying on neuroendocrine pathway regulation [40]. Follow-up experiments are planned to assess changes in plasma hormone levels, like adrenocorticotropic hormone (ACTH)/corticotropin-releasing hormone (CRH**),** after CNO intervention using enzyme-linked immunosorbent assay (ELISA) techniques to exclude endocrine pathway effects. However, the specific neurotransmitter phenotype of the PVN-preBötC circuit and its regulatory mechanisms remain unclear. Previous studies indicate that glutamate is a key neurotransmitter in the PVN, with most glutamate-expressing neurons identified using the vesicular glutamate transporter 2 (VGLUT2) marker [41]. Future experiments may involve retrograde tracing, single-cell nuclear RNA sequencing (snRNA-seq), and multiple fluorescence *in situ* hybridization (MERFISH) to quantify the subpopulation of PVN neurons projecting to preBötC and clarify the co-expression proportions of VGLUT2/CRH/OXT in PVN. Fiber optic photometry could be used to synchronize the recording of transmitter release dynamics after optogenetic activation of PVN by implanting genetically encoded GPCR-activation-based(GRAB)sensors in preBötC to elucidate the relationship between neurotransmitter release, such as glutamate, and respiratory rhythms.

The findings of this study support a working model in which neurons in the PVN respond to CIH by increasing neuronal projections to the preBötC *via* the PVN-preBötC circuit. This response accelerates the respiratory rate, enhancing basal ventilation. However, the specific mechanisms underlying this respiratory regulation require further investigation. The specific neurons and phenotypes involved in respiratory regulation *via* PVN projections in the context of CIH remain unknown. This study did not rule out the potential direct or indirect involvement of the PVN in regulating respiration through other pathways. This will be one of the key areas of focus in our subsequent research. The next step involves collecting 7 T cranial MRIs from patients with CIH, including those with OSAHS, to further validate this working model and integrate it with clinical disease models. In the future, a comparative analysis will determine whether there are differences in the morphology and metabolomics of the PVN brain regions between normal and CIH environments.

In conclusion, this study provides evidence of a direct relationship between the PVN and the preBötC. It anatomically clarified the importance of the PVN within the respiratory neural network and established a direct projection from the preBötC to the spinal phrenic nucleus. This lays the groundwork for future research to further elucidate the neural pathways connecting the spinal respiratory motor nerves with upstream nuclei. Furthermore, this study revealed that PVN activation in the CIH environment likely contributes to respiratory control *via* projections in the PVN-preBötC circuit, influencing basal MV by modifying the RF. These insights may offer new targeted therapeutic approaches for hypoxia-related diseases such as OSAHS. For instance, transcranial stimulation of specific neuronal populations could enable respiratory pacing therapy, addressing respiratory ventilation issues at the neural circuit level to resolve hypoxia.

## Supplementary Material

ARRIVE_Checklist.pdf

## Data Availability

The data generated by this research can be obtained from the author Linglin Liu upon reasonable request.
